# Schwannomas mistaken for metastases of melanoma in PET‐CT: A diagnostic challenge with consequences

**DOI:** 10.1002/ccr3.6753

**Published:** 2022-12-27

**Authors:** Stefanie Hirsiger, Radu Olariu

**Affiliations:** ^1^ Department of Plastic and Hand Surgery, Inselspital University Hospital Bern University of Bern Bern Switzerland

**Keywords:** cutaneous malignant melanoma, FDG‐PET, metastasis, schwannoma

## Abstract

The incidence of skin cancer and especially cutaneous malignant melanoma is rising, as are indications for staging examinations to detect metastasis. Schwannomas are common benign nerve tumors, which can be mistaken for metastasis even in highly specialized imaging. Risk of nerve lesion is high in inadvertent biopsy.

## Graphical abstract

Schwannoma should be kept in mind as a differential diagnosis for metastatic melanoma as it similarly shows high intensity in FDG‐PET‐CT. The main risk of surgical and CT‐guided biopsies is nerve injury. Functional diffusion tensor imaging might help to differentiate schwannoma from melanoma or sarcoma metastasis.

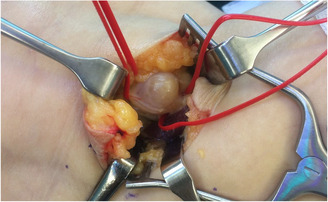

## 1 INTRODUCTION

The incidence of invasive melanoma continues to increase in Europe.^1^ Although survival in metastatic melanoma has been markedly increased thanks to major breakthroughs in therapy, it is still a potentially lethal illness.^2^ The diagnosis of metastatic disease is crucial for guiding systemic therapy.^3^ Fluordesoxyglucose positron emission tomography with computed tomography (FDG‐PET‐CT) is useful in the staging of cutaneous malignant melanoma (CMM)^4^ and is especially helpful in advanced disease or when suspecting recurrence.^5^ Other malignant skin tumors like dermal sarcomas are very rare but occurred in one of the clinical cases described below.^6^ Although FDG‐PET‐CT can often help to distinguish between malignant and benign lesions,^7^ some high‐uptake lesions are benign. The latter is especially true for schwannoma, a benign encapsulated tumor of the peripheral nervous system arising from the Schwann cells in the nerve sheath. If metastasis is suspected in FDG‐PET‐CT, histologic confirmation by image‐guided or surgical biopsy is usually indicated for confirmation. The differential diagnosis of schwannoma is important, as percutaneous and even open surgical biopsies carry a risk of associated neurologic lesion.^8^ In our unit, we treated two patients with melanoma and one patient with pleomorphic dermal sarcoma and suspected lymphatic metastasis in FDG‐PET‐CT, which were diagnosed as schwannomas on histologic evaluation of the biopsies. One patient suffered neurologic lesions following the biopsy.

## 2 CASE PRESENTATION

### 2.1 Case 1

A 66‐year‐old patient was diagnosed with CMM in the right scapular area. After excisional biopsy, the histology showed a Breslow depth of 5 mm with ulceration and satellite nodes. A computer tomography revealed no suspicion of metastasis at the time leading to an initial classification of pT4b pN2c cM0, AJCC‐stage IIIC in November 2012. Three years later during follow‐up, a lumbar cutaneous melanoma metastasis was excised and histologically confirmed.

**FIGURE 1:**
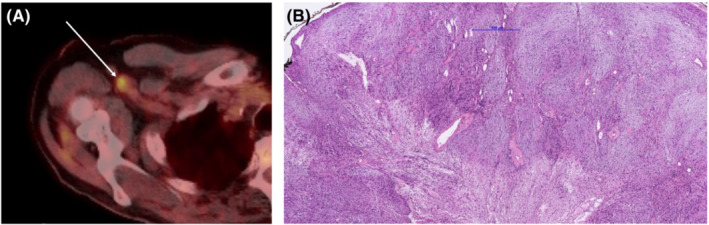
(A) FDG‐PET‐CT with high‐uptake right axillary lesion. (B) Histologic overview of the lesion showing typical features of schwannoma with hyper‐ and hypocellular areas.

For staging, the patient had a FDG‐PET‐CT that showed a suspicious lesion in his right axilla (Figure 1) with recommendation of excision and histopathologic evaluation by the interdisciplinary skin cancer tumor conference. Excision was performed and histologic examination showed a schwannoma (Figure 1B). Unfortunately, partial nervous injury occurred during surgery causing neuropathic pain of the I–III finger and paresis of flexor pollicis longus and flexor digitorum profundus dig. II muscles.

Two years later in 2017, 5 years after the initial diagnosis, an FDG‐PET‐CT showed a 5‐mm subcutaneous lesion in the left gluteal area, which was excised and showed an angiolipoma. In 2019, the patient was diagnosed with a lumbar melanoma metastasis and is currently treated for metastatic disease with immune therapy (Pembrolizumab 2 mg/kg body weight i.v. every 3 weeks).

The neurologic deficit has improved over time, but slight sensory and motor impairment is persistent with a Tinel sign along the median nerve, atrophy of the thenar muscles, but a good pinch force of 5 kg versus 6 kg on the healthy contralateral side.

### 2.2 Case 2

**FIGURE 2:**
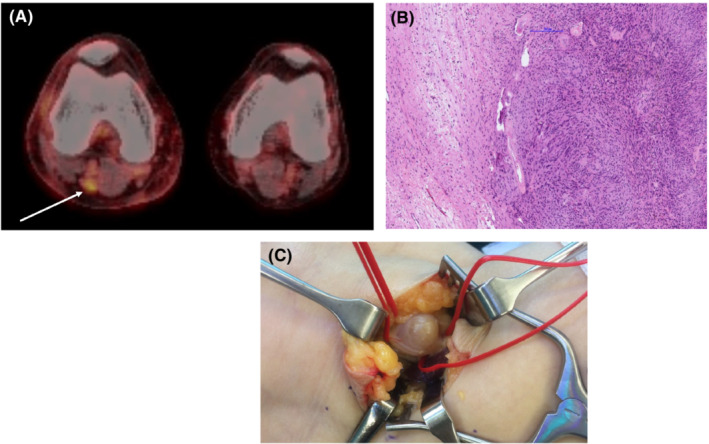
(A) FDG‐PET‐CT with high‐uptake right popliteal lesion. (B) Antoni A areas (hypercellular, with palisading) and Antoni B areas (hypocellular, myxoid) are characteristic for schwannoma. (C) Intraoperative suspicion of schwannoma

A 67‐year‐old patient was diagnosed with a CMM of the superficial spreading type of the dorsal foot in August 2017. Excision showed a Breslow of 2.35 mm, Clark level IV with ulceration. An excision with safety margin and inguinal SLNB was conducted with confirmation of an inguinal lymphatic metastasis. At that time, the lymphoscintigraphy showed only inguinal SLNs. The initial classification was pT3b pN1a cM0, AJCC‐stage IIIC. Seven days postoperatively, an ultrasound was conducted for swelling of the leg to exclude a thrombosis. No thrombosis, but a popliteal tumor suspicious for lymph node metastasis was described by the radiologists. An FDG‐PET‐CT confirmed a popliteal high‐uptake tumor, interpreted as a pathologic lymph node suspect for metastasis or malignancy with recommendation of excision (Figure 2A). Surgery and complete open excision of a perineurally located tumor along the superficial sural nerve was performed 3 weeks later, histologic analysis revealing schwannoma (Figure 2B). The sural nerve could be spared since the intraoperative situation was clinically highly suspicious of a schwannoma (Figure 2C). Postoperatively, the patient is asymptomatic concerning all potentially involved nerves.

### 2.3 Case 3

An 80‐year‐old patient was operated for a CMM of superficial spreading type with nodular growth on the left upper arm in June 2010. Excision and SLNB was performed and showed a Breslow 1.42 mm without ulceration, Clark level IV and no lymphatic metastasis in June 2010. The initial classification was pT2a pN0 cM0, AJCC‐stage IB.

**FIGURE 3:**
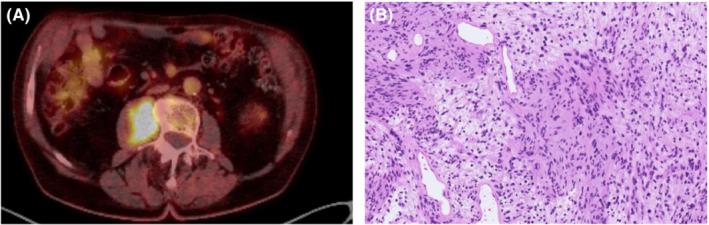
(A) FDG‐PET‐CT with high uptake in right psoas muscle lesion. (B) Image‐guided core‐needle biopsy showing same typical morphology of schwannoma: intermingled Antoni A and B areas.

One year after the melanoma diagnosis, in 2011, a pleomorphic dermal sarcoma (PDS) of the left shoulder was found, which was resected in toto. In 2017, he developed a recurrence of the PDS, which was confirmed by biopsy before wide excision with 2 cm tissue margins. A staging PET‐CT showed a tumor in the psoas muscle (Figure 3A), highly suspect for metastasis and an MRI confirmed the suspicion. Image‐guided biopsy of the tumor deemed the suspicion wrong and revealed the histologic diagnosis of a schwannoma, confirmed by immunohistochemical staining in 12/2017 (Figure 3B). Post intervention, the patient did not suffer from any neuropathic pain or deficits.

## 3 DISCUSSION/CONCLUSION

We present two patients who have been operated for suspected melanoma lymph node metastasis and one patient with pleomorphic dermal sarcoma (and melanoma in the past), which all turned out to be asymptomatic benign schwannomas. In one patient, the surgical procedure had significant adverse effects in form of functional deficit and persistent neuropathic pain. If the differential diagnosis of schwannoma had been considered preoperatively, the surgery could have been performed with more attention to the nerve involved and injury might have been prevented. The differential diagnosis between malignant and benign lesions is not always possible by FDG‐PET‐CT, which is especially true in the case of schwannomas.^9^, ^10^

Magnetic resonance imaging can often help to localize the tumor in association with a nerve, but most of the times the exact tumor type cannot be differentiated.^11^ To help differentiate between malign and benign lesions, functional diffusion tensor imaging can be performed as a valuable adjunct. The latter allows fiber tracking and whereas complete disruption of nerve fibers in tractography and low diffusivity values suggests malignancy in neural masses, orderly fibers, and nerve continuity are suggestive for schwannoma.^12^

Due to the increased incidence of melanoma and the widespread use of FDG‐PET‐CT for staging, the incidence of false‐positive lesions and thus nerve lesions due to inadvertent biopsy of schwannoma might increase.

In conclusion, schwannoma should be kept in mind as a differential diagnosis for metastatic lesions of melanoma that show high intensity in FDG‐PET‐CT. The main risk of surgical but also image‐guided biopsies is symptomatic nerve injury. Recent developments in imaging are promising, but no gold standard has yet been defined. In the future, functional diffusion tensor imaging might help to differentiate schwannoma from metastasis or other malignant tumors in uncertain cases.

## AUTHOR CONTRIBUTIONS

**Stefanie Hirsiger:** Visualization; writing – original draft; writing – review and editing. **Radu Olariu:** Conceptualization; supervision; validation; writing – review and editing.

## FUNDING INFORMATION

This research received no specific grant from any funding agency in the public, commercial, or not‐for‐profit sectors.

## CONFLICT OF INTEREST

All authors hereby declare that they have no potential conflicts of interest to disclose.

## CONSENT

An approval of the ethics committee is not required for case series according to Swiss law. Written informed consent was obtained from the patient to publish this report in accordance with the journal's patient consent policy.

## Data Availability

Data available on request from the authors.

## References

[1] Sacchetto L , Zanetti R , Comber H , et al. Trends in incidence of thick, thin and in situ melanoma in Europe. Eur J Cancer. 2018;92:108‐118. 29395684 10.1016/j.ejca.2017.12.024

[2] Pasquali S , Hadjinicolaou AV , Chiarion Sileni V , Rossi CR , Mocellin S . Systemic treatments for metastatic cutaneous melanoma. Cochrane Database Syst Rev. 2018;2:CD011123. 29405038 10.1002/14651858.CD011123.pub2 PMC6491081

[3] Swetter SM , Tsao H , Bichakjian CK , et al. Guidelines of care for the management of primary cutaneous melanoma. J Am Acad Dermatol. 2019;80(1):208‐250. 30392755 10.1016/j.jaad.2018.08.055

[4] Krug B , Crott R , Lonneux M , Baurain JF , Pirson AS , Vander BT . Role of PET in the initial staging of cutaneous malignant melanoma: systematic review. Radiology. 2008;249(3):836‐844. 19011184 10.1148/radiol.2493080240

[5] Perissinotti A , Rietbergen DD , Vidal‐Sicart S , Riera AA , Olmos RAV . Melanoma & nuclear medicine: new insights & advances. Melanoma Manag. 2018;5(1):MMT06. 30190932 10.2217/mmt-2017-0022 PMC6122522

[6] Ibanez MA , Rismiller K , Knackstedt T . Prognostic factors, treatment, and survival in cutaneous pleomorphic sarcoma. J Am Acad Dermatol. 2020;83(2):388‐396. 30414918 10.1016/j.jaad.2018.08.054

[7] Watanabe H , Shinozaki T , Yanagawa T , et al. Glucose metabolic analysis of musculoskeletal tumours using 18fluorine‐FDG PET as an aid to preoperative planning. J Bone Joint Surg. 2000;82(5):760‐767. 10.1302/0301-620x.82b5.9824 10963181

[8] Siqueira MG , Socolovsky M , Martins RS , et al. Surgical treatment of typical peripheral schwannomas: the risk of new postoperative deficits. Acta Neurochir. 2013;155(9):1745‐1749. 23873125 10.1007/s00701-013-1818-6

[9] Aoki J , Watanabe H , Shinozaki T , et al. FDG‐PET for preoperative differential diagnosis between benign and malignant soft tissue masses. Skeletal Radiol. 2003;32(3):133‐138. 12605276 10.1007/s00256-002-0586-9

[10] Beaulieu S , Rubin B , Djang D , Conrad E , Turcotte E , Eary JF . Positron emission tomography of schwannomas: emphasizing its potential in preoperative planning. AJR Am J Roentgenol. 2004;182(4):971‐974. 15039173 10.2214/ajr.182.4.1820971

[11] Nilsson J , Sandberg K , Soe Nielsen N , Dahlin LB . Magnetic resonance imaging of peripheral nerve tumours in the upper extremity. Scand J Plast Reconstr Surg Hand Surg. 2009;43(3):153‐159. 19401939 10.1080/02844310902734572

[12] Chhabra A , Thakkar RS , Andreisek G , et al. Anatomic MR imaging and functional diffusion tensor imaging of peripheral nerve tumors and tumorlike conditions. AJNR Am J Neuroradiol. 2013;34(4):802‐807. 23124644 10.3174/ajnr.A3316 PMC4629840

